# The effects of non-diagnostic information on confidence and decision making

**DOI:** 10.3758/s13421-024-01535-6

**Published:** 2024-03-15

**Authors:** Amelia T. Kohl, James D. Sauer, Matthew A. Palmer, Jasmin Brooks, Andrew Heathcote

**Affiliations:** 1 School of Psychology, University of Birmingham, Birmingham, B15 2TT UK; 2 School of Psychological Sciences, University of Tasmania, Tasmania, Australia; 3 School of Psychological Sciences, The University of Newcastle, Newcastle, Australia

**Keywords:** Confidence, Decision making, Doubt scaling, Evidence accumulation

## Abstract

Many decision-making tasks are characterized by a combination of diagnostic and non-diagnostic information, yet models of responding and confidence almost exclusively focus on the contribution of diagnostic information (e.g., evidence associated with stimulus discriminability), largely ignoring the contribution of non-diagnostic information. An exception is Baranski and Petrusic’s *Journal of Experimental Psychology: Human Perception and Performance, 24*(3), 929-945, (1998) doubt-scaling model, which predicts a negative relationship between non-diagnostic information and confidence, and between non-diagnostic information and accuracy. In two perceptual-choice tasks, we tested the effects of manipulating non-diagnostic information on confidence, accuracy and response time (RT). In Experiment 1, participants viewed a dynamic grid consisting of flashing blue, orange and white pixels and indicated whether the stimulus was predominantly blue or orange (using a response scale ranging from low-confidence blue to high-confidence orange), with the white pixels constituting non-diagnostic information. Increasing non-diagnostic information reduced both confidence and accuracy, generally slowed RTs, and led to an increase in the speed of errors. Experiment 2 replicated these results for a decision-only task, providing further support for the doubt-scaling model of confidence.

## Introduction

We examine what we believe to be an empirically and theoretically interesting and important, but somewhat neglected, question: How are decision confidence, speed and accuracy affected by ‘non-diagnostic’ information (i.e., information that is not relevant to the choice and hence not determinative of accuracy)? Many theories of choice assume that relevant evidence drives the decision process. This is true by construction when the evidence is unidimensional, as is assumed by the most widely applied theory of binary choice accounting for accuracy, Signal Detection Theory (SDT; Bernbach, 1971; Egan et al., 1959; Green & Swets, 1966). It is also true of the most widely applied dynamic theory of binary choice, which also accounts for accuracy and response time (RT), the Diffusion Decision Model (DDM; Ratcliff & McKoon, 2008). In both cases the input to the decision process is constructed from the evidence for one choice option minus the evidence for the other choice option, so the effect of any non-diagnostic information is effectively ignored. Although both frameworks account decision making, they have not been extended to make explicit predictions about the effects of non-diagnostic information on RT and accuracy.

However, another widely applied class of dynamic choice theories – accumulator models (Audley, 1960, Brown & Heathcote, 2008, Tillman et al., 2020, Usher & McClelland, 2001, Vickers, 1970; Smith & Vickers, 1988: Vickers & Lee, 1998, Van Zandt et al., 2000) – have separate inputs corresponding to each response option, and could more easily allow for explicit predictions regarding the effects of non-diagnostic information. Baranski and Petrusic’s (1998) doubt-scaling model comes from this class, and is the only theory we know of quantitatively predicting accuracy and RT that attempts to directly address the effects of non-diagnostic information. Their model also addresses decision confidence, and it is with respect to confidence that there has been the most interest in the effects of non-diagnostic information. This interest is motivated by dissociations between confidence and accuracy – running counter to the generally robust positive confidence-accuracy relationship – that have been attributed to non-diagnostic information (e.g., Busey et al., 2000; Manley et al., 2019). However, empirical studies of confidence have rarely included direct quantitative manipulations of non-diagnostic information. In this paper we study a perceptual choice task that affords such a direct manipulation, and use our results to test predictions of the doubt-scaling model. Before reporting the results of two experiments, we first discuss why the effects of non-diagnostic information are of both theoretical and applied interest, describe the doubt-scaling model, and derive from it predictions about accuracy, RT and confidence.

## The doubt-scaling model of confidence

The positive confidence-accuracy relationship has been influential in both theoretical and applied domains (Brewer & Wells, 2006; Gigerenzer et al., 1991; Juslin et al., 2000; Palmer et al., 2013; Sauer et al., 2010). For example, in eyewitness identification, confidence is often relied upon to assess the reliability of a decision when the correct response is unknown (Brewer & Wells, 2006; Wixted & Wells, 2017; National Academy of Sciences (NAS), 2014). However, factors unrelated to accuracy may also shape confidence (Busey et al., 2000; Baranski & Petrusic, 1998; Van Zandt, 2000). For example, Busey et al. found that confidence in face recognition decisions increased, with no corresponding increase in accuracy, when the luminance of an image increased from study to test. Accuracy, however, was improved when luminance at encoding was test matched. Hence, non-diagnostic information can inflate confidence.

Although the doubt-scaling model is alone in explicitly accounting for the effect of non-diagnostic information on accuracy, RT and confidence, it has not, to our knowledge, been directly tested. Understanding the role of non-diagnostic information on confidence, accuracy and RT could be of great value in applied settings, where decision stimuli often contain non-diagnostic information. For example, consider an eyewitness identification test. During the commission of a crime, parts of a perpetrator’s face may be concealed from or unobserved by an eyewitness. However, when the witness later views a lineup, the faces of the lineup members may be presented unobstructed. Thus, each face will contain featural and configural information that is non-diagnostic because that information was not encoded during the initial event and therefore cannot contribute to genuine recognition.

For example, Manley et al. (2019) conducted a face-recognition task involving a combination of full and partial faces (faces where only the eye area was visible, as might be the case if the perpetrator was wearing a ski-mask). Participants’ confidence in their recognition decisions was lower for trials in which they studied a partial face but were tested with a full face, suggesting that decision confidence was reduced by the additional, non-diagnostic information present at test (i.e., parts of the face obscured at study but visible at test). Given the recent international surge of mask-wearing for health reasons, it is important to understand how non-diagnostic information affects recognition, and the confidence and RT associated with recognition. Non-diagnostic information may also affect applied perceptual discrimination tasks. For example, when border security agents compare passport images to real faces, some features are relatively stable and therefore likely to be diagnostic (e.g., shape of the face, distance between eyes), while others are easily changeable and therefore may prove non-diagnostic (e.g., colour/length of hair, lighting). Understanding how non-diagnostic information affects decision-making processes may have substantial applied value.

Although it could be argued that non-diagnostic information is simply a source of noise, the doubt-scaling model provides an alternative hypothesis. While it is generally accepted that noise impairs the decision-maker’s ability to appraise diagnostic information (therefore indirectly affecting confidence by interfering with the evaluation of information that underlies such judgements), the doubt-scaling model instead suggests that non-diagnostic information is central to the assessment of confidence. Thus, the aim of this study was to empirically determine the veracity of this claim.

The doubt-scaling model of confidence (Baranski & Petrusic, 1998) evolved from the slow-and-fast guessing theory (Petrusic, 1992). It is an extension of a very early type of evidence-accumulation model, Audley’s (1960) ‘runs model’ of binary choice, which assumes that on each time step evidence is dichotomized as either A > B (favouring choice A) or B > A (favouring choice B). This discrete evidence is tallied in corresponding accumulators until a response threshold is reached, triggering a decision. Slow-and-fast guessing theory proposes a third accumulation process: A = B (i.e., non-diagnostic evidence favouring neither choice option). If the A = B accumulator reaches its threshold first a guess response is triggered, reducing accuracy. The doubt-scaling model expands upon this account by making explicit predictions regarding the relationship between non-diagnostic information and confidence; specifically, that confidence is inversely proportional to the amount of information accumulated for A = B. Thus, the more non-diagnostic information accumulated, the less confident the responder will be.

Rather than testing the doubt-scaling model quantitively, and hence having to commit to all of the specific details of the runs model, we instead tested more general ordinal predictions made by the model. First, the presence of non-diagnostic information slows overall RT. Accumulation in the runs model is competitive (as is assumed by more modern evidence-accumulation models, e.g., Usher & McClelland, 2001; van Ravenzwaaij et al., 2020): if one type of evidence is tallied on a given time-step, the other evidence totals remain unchanged. Hence, when evidence increments are shared among accumulators, the time for any one accumulator to reach threshold is slowed.

**Fig. 1:**
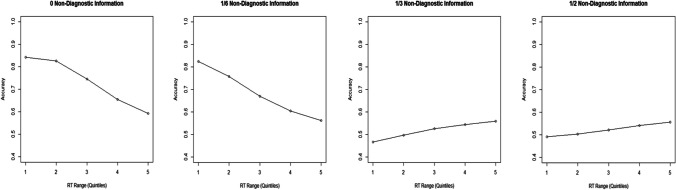
Conditional-accuracy functions (CAFs) of model simulated data, illustrating a ‘flattening’ of the curve as the proportion of non-diagnostic information increases (due to a decrease in accurate responses in the faster response time (RT) bins). *Note:* CAFs of simulated data are from an adapted version of Audley’s (1960) runs model (incorporating an additional accumulator for non-diagnostic information that, in turn, drives ‘guessing’ responses. R code available at: https://osf.io/exqba/). Accuracy is shown as a function of RT. The first point on the x-axis represents the fastest responses (below the 20th percentile of RT), the second point the 20th to 40th percentile, etc

A more fine-grained prediction is made with respect to the relationship between RT and accuracy: the speed and frequency of guessing responses increases as the rate of A = B increments increases. We cannot observe guessing and non-guessing responses separately, but we can compare the speed of less accurate responses (which should include more guessing responses) and more accurate responses (which should include more non-guessing responses) using Conditional Accuracy Functions (CAFs; Thomas, 1974; Elliott et al., 2022). CAFs plot accuracy as a function of RT, with responses being ordered by RT and grouped into a series of equal-sized ‘bins’ (e.g., the fastest 20% of responses, the next fastest 20%, etc.) within which accuracy is calculated. The doubt-scaling model predicts that as overall accuracy decreases with an increase in non-diagnostic information, the accuracy of responses in the faster bins will decrease relative to the accuracy of the slower bins, resulting in a flattening of the CAF (see Fig. 1).

## The current study

Due to the lack of explicit consideration given to the role of non-diagnostic information in the decision literature, we have endeavored to empirically test the predictions of the doubt-scaling model (1998), which suggests that non-diagnostic information has an important role in driving decision making (rather than simply a source of noise in the evidence-accumulation process). In the first of two experiments, we investigate the effects of differing levels of non-diagnostic information on confidence, accuracy and RT in a perceptual task. In the second, we look at the effects of non-diagnostic information solely on accuracy and RT.

## Experiment 1

The choice stimuli in Experiment 1 were grids containing a combination of blue, orange and white pixels the arrangement of which changed dynamically. Participants provided a decision, and a confidence rating for their decision, about whether there were more orange or blue pixels, with an equal number of trials where blue pixels outnumbered orange pixels compared to trials where orange pixels outnumbered blue. Hence, the white pixels provided non-diagnostic information.

We introduced two manipulations that test the generality of the doubt-scaling model’s predictions, one of which provides a further test of the model. First, the more common colour constituted 55% of the diagnostic pixels in an easy-choice condition and 52% in a hard-choice condition. This difficulty factor was crossed with two ways of manipulating the amount of non-diagnostic information. In all cases there were four levels of non-diagnostic information, a control condition with no white pixels, and low, moderate and high non-diagnosticity conditions, where white pixels constituted, respectively, one-sixth, one-third or one-half of the total number of pixels. In the ‘additive’ manipulation, the number of coloured (i.e., diagnostic) pixels was kept constant as the number of white pixels increased, leading to an increase in total grid size. In the ‘stable’ condition, grid size remained constant, and the total number of diagnostic pixels decreased while maintaining the blue-to-orange proportion. If the absolute amount of diagnostic or non-diagnostic information is important, the results for these two manipulations could differ. In contrast, the runs model assumes that all that matters is the relative amounts of the three different types of information, so the doubt-scaling model predicts no difference between the additive and stable conditions.

In summary, we expected that accuracy and confidence would be less, and RT slower, for the hard condition than for the easy condition. The doubt-scaling model predicts that as non-diagnostic information increases, in both the hard and the easy conditions, confidence will decrease and RT will increase; that the accuracy of faster responses will decrease relative to slower responses; and that the additive and stable conditions will not differ. If the absolute amount of non-diagnostic information is important, we would expect our manipulation of non-diagnostic information to have a larger effect in the additive condition. Alternatively, if the absolute amount of diagnostic information is more influential, we would expect a larger effect in the stable condition (where any increase in non-diagnostic information involves a corresponding decrease in diagnostic information).

### Method

#### Design

We used a 2 (grid-type: stable or additive) × 4 (proportion of non-diagnostic information: 0, 0.17, 0.33, 0.50) × 2 (difficulty: easy vs. hard) × 2 (majority colour: blue vs. orange) mixed design, with grid-type as the between-subjects factor. Dependent variables are decision confidence (low, moderate, and high), mean RT, and accuracy; measured by the equal-variance signal-detection theory discrimination (*d´*) measure, and proportion correct as a function of RT (as used in the CAFs).

#### Participants

We randomly allocated 56 participants to the stable or additive condition, so as to allow for a minimum of 20 participants per cell. Eight participants were excluded from analyses as their data showed truncated RT distributions due to the 5-s response window (n = 5), or below 55% accuracy on ‘easy’ trials (n = 3). An additional participant was excluded for incomplete data. This left 22 participants in the stable condition and 25 participants in the additive condition. First-year psychology students were reimbursed with research credits and other participants received a $20 e-voucher. Participants were required to have normal or corrected-to-corrected normal vision, and were not eligible to participate if they suffered from epilepsy or related conditions.

#### Materials

**Fig. 2:**
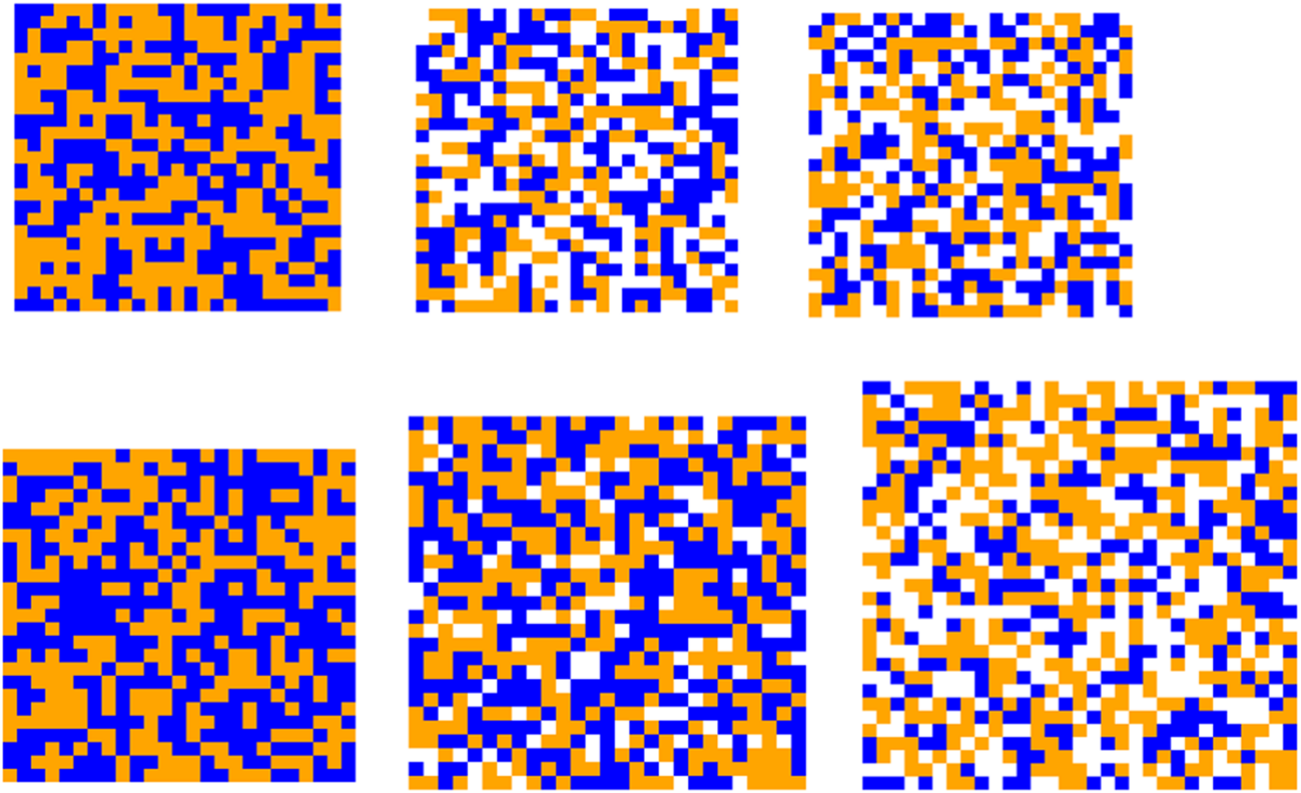
Schematic representations of the dynamic-grid stimulus at varying levels of non-diagnostic information. White pixels represent non-diagnostic information, and the total proportion of non-diagnostic information increases from left to right. The first row represents the stable condition, whereas the second row represents the additive condition

Participants completed the task on in-lab desktop computers equipped with 3.30 GHz Intel i5-6600 processors, 16 GB RAM, and a Windows 7 enterprise operating system configured to minimize internal task-switching. The program was written and run using MATLAB (The MathWorks, R2016b). For each trial, participants viewed a dynamic grid consisting of blue (RGB = 0, 65, 255), orange (RGB = 255, 127, 0) and sometimes white (RGB = 128, 128, 128) pixels (see Fig. 2). Although the colour of pixels in the grid changed constantly, the proportion of blue, orange and white pixels remained constant. Table 1 provides a breakdown of how the coloured pixels varied between different levels of non-diagnostic information for each grid type.

Participants responded by moving their mouse from the start point (a circle on the screen, equidistant from six response options) to the relevant segment of the response arc (labelled low-confidence blue, moderate-confidence blue, high-confidence blue, low-confidence orange, moderate-confidence orange and high-confidence orange). This design allows RT data for multiple levels of confidence to be collected in a way that minimizes noise associated with motor responses (e.g., differences in motor time associated with the use of different fingers to indicate confidence using a keyboard). Participants clicked inside a circle in the middle of the screen to begin each trial. Participants who responded too quickly (before .15 s) were warned that they were too fast. Participants who took longer than 2 s to respond were warned that they were responding too slowly. If participants did not respond within 5 s, the trial ended, and they received an on-screen message saying they were too slow to respond.

In the stable condition, the overall grid size remained constant. Thus, as the overall proportion of non-diagnostic pixels (i.e., white pixels) increased, the number of diagnostic pixels (i.e., blue and orange pixels) decreased. In the additive condition, the dynamic grid increased in size as the proportion of non-diagnostic pixels increased, meaning the number of diagnostic pixels remained constant (529 diagnostic/coloured pixels). In easy trials, 55% of the diagnostic pixels consisted of the dominant colour (i.e., correct response). In hard trials, 52% of the diagnostic pixels consisted of the dominant colour (i.e., correct response). The remainder of the grid was filled with the incorrect colour, and non-diagnostic pixels.

#### Procedure

Participants first completed three training blocks, with the first two blocks comprising 20 trials and the third block 40. In all three practice blocks, participants were provided with feedback indicating whether each response was correct. In the first practice block, participants responded to a stimulus like that in Fig. 1 (i.e., pixels were either orange or blue; no white was included), by simply indicating whether the stimulus was predominantly orange or blue, with no confidence ratings required. The second practice block introduced the manipulation of non-diagnostic information (i.e., white pixels), and the third practice block introduced the six response categories (high-confidence blue, moderate-confidence blue, etc.). This approach was intended to help participants learn the demands of the task before starting experimental trials. Each experimental block comprised 80 trials. Participants completed nine experimental blocks. Unlike the practice blocks, participants did not receive feedback for the experimental trials. Participants were encouraged to take rests between blocks as required. The task took approximately 1 h to complete.

#### Analysis methods

Few participants consistently used all three levels of confidence, with participants varying in the least-used level. To produce stable estimates for our analyses, we collapsed responses to two levels of confidence (‘low’ and ‘high’). For each participant, moderate responses were collapsed into either the high or low category based on upon which of these two options was used less frequently.

We used linear mixed-effect models assuming Gaussian error to analyze the logarithm of RT and generalized linear mixed-effect models with a probit link function to analyze the probability of high-confidence responses (Bates et al., 2015; Kuznetsova et al., 2017). Participant was set as a random factor, with grid-type (additive/stable), proportion of non-diagnostic information (0, 0.17, 0.33, 0.50), difficulty (hard/easy), and predominant stimulus colour (orange or blue) included as fixed effects.

Due to response bias in the data – participants showed a bias towards orange stimuli at low proportions of non-diagnostic information, and a bias towards blue stimuli at high levels of non-diagnostic information – we analyzed accuracy using the SDT-based measure of discrimination, *d´*, rather than raw accuracy scores. This allowed us to determine the effect of non-diagnostic information on participants’ ability to discriminate between the correct and incorrect response independent of response bias. The discrimination analysis was accomplished using a generalized linear mixed-effect model with a probit link function on the proportion of blue responses, with *d*´ corresponding to the difference between majority blue versus majority orange stimuli, and effects on *d*´ corresponding to interactions with the stimulus factor.

We constructed CAFs by dividing responses into quintile bins (separately for the stable and additive conditions, and then collapsed across both). For the first bin the accuracy of the fastest 20% of responses is plotted, for the second the accuracy for RTs between the 20th and 40th percentiles and so on up to the slowest 20% of responses for the fifth bin. The choice of number of bins is arbitrary; this relatively coarse division results in precise accuracy estimates for each bin as they are based on many responses. The same pattern of results, albeit with more variability, was found using more bins.

## Results

Statements about significance are made with respect to a .05 criterion. Although we included the majority-colour factor in the RT and confidence ANOVAs we do not report tests of it as they are not germane to our hypotheses. Full ANOVA tables for confidence, accuracy and RT are available on OSF (available at https://osf.io/exqba/) and important effects are summarized below.

### Confidence

Consistent with the doubt-scaling model, the proportion of high-confidence responses decreased as the amount of non-diagnostic information increased, χ^2^(3) = 2102.32, *p* < .001 (see Fig. 2, panels A and B). There was also a significant main effect of difficulty on confidence, with easy trials receiving a higher proportion of high-confidence responses than hard trials, χ^2^(1) = 95.64, *p* < .001, with no significant interaction between the two effects (see Fig. 2). Although the main effect of grid type, and interactions with difficulty, were non-significant, grid type did interact with the proportion of non-diagnostic information, χ^2^(1) = 24.25, *p* < .001. However, the interaction effect was only small: non-diagnostic information exerted a slightly greater effect on confidence in the stable condition (mean confidence decreased from 62% at zero non-diagnostic information to 29% at half non-diagnostic information) than in the additive condition (59% vs. 31%), collapsing across difficulty conditions.

### Discrimination

**Fig. 3:**
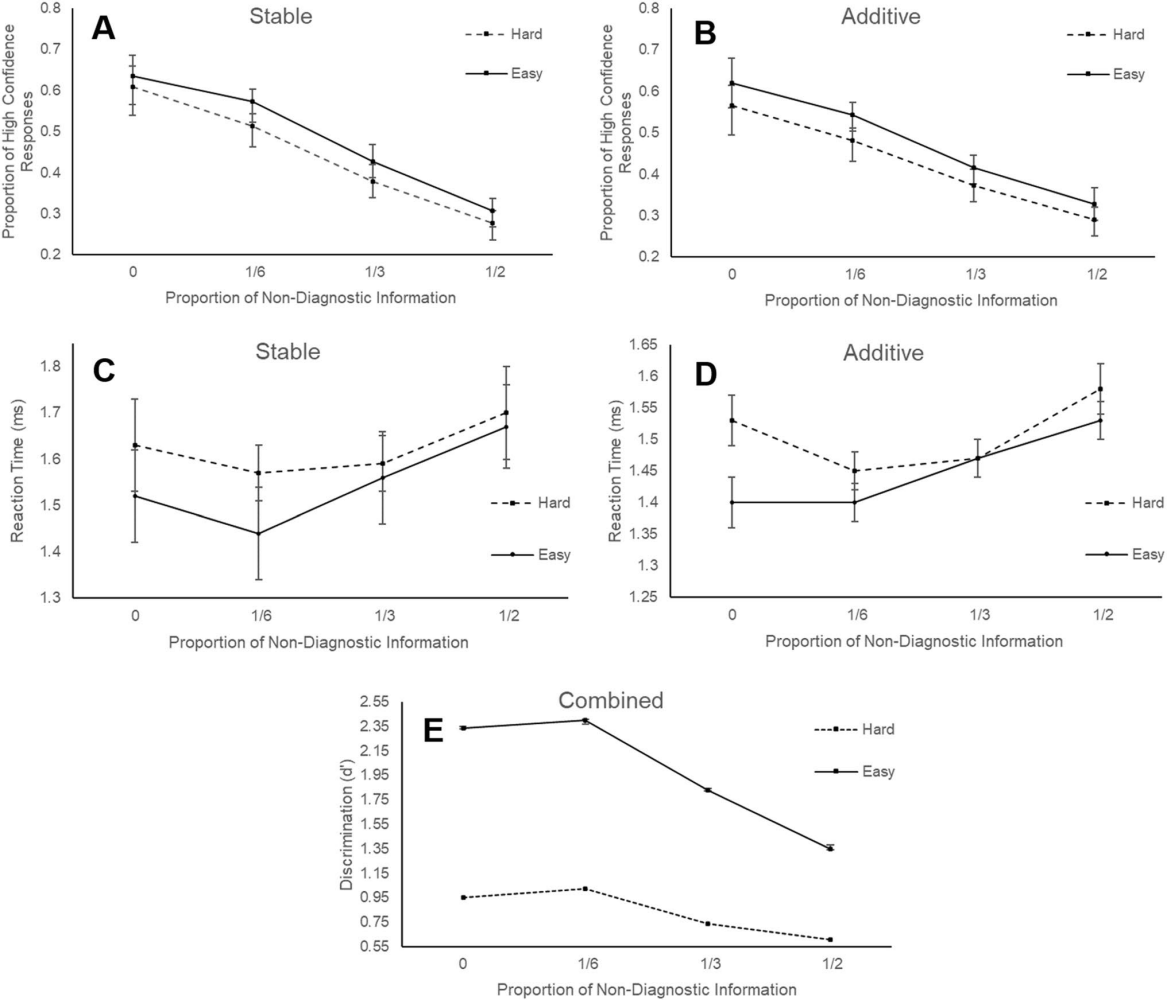
The effects of non-diagnostic information on confidence, accuracy (*d´*) and response time (RT) for Experiment 1. Figures demonstrate the relationship between non-diagnostic information and confidence (panels **A** and **B**), RT for all responses (panels **C** and **D**), and accuracy (as indexed by d´, panel **E**). Error bars represent the standard error

Increasing the proportion of non-diagnostic information also affected discrimination, χ^2^(3) = 322, *p* < .001. As shown in Fig. 3 (panel E), discrimination was nearly identical at the two lowest levels, but decreased systematically thereafter. Difficulty had the expected strong main effect, χ^2^(1) = 1251, *p* < .001, and the amount of non-diagnostic information had a weaker effect for hard than for easy trials, χ^2^(1) = 66.8, *p* < .001. The only effect including grid type was a relatively weak interaction where the difficulty effect was larger in the stable than in the additive condition, χ^2^(1) = 4.83, *p* = .03.

#### Response times

RT generally slowed as the proportion of non-diagnostic information increased, *F*(3,33599) = 109.7, *p* <.001, and was faster for easy than for hard choices, *F*(3,33599) = 218.2, *p* < .001. However, as shown in Fig. 3 (panels C and D), the slowing was restricted to conditions where there was some non-diagnostic information, and the effect of non-diagnostic information was again weaker for the hard condition than for the easy condition. The only significant effect of grid type was a significant interaction with both difficulty and non-diagnostic information, *F*(3,33599) = 2.95, *p* =.03, due to a larger difficulty effect for stable than additive for the low proportion of non-diagnostic information.

#### Conditional accuracy functions

**Fig. 4:**
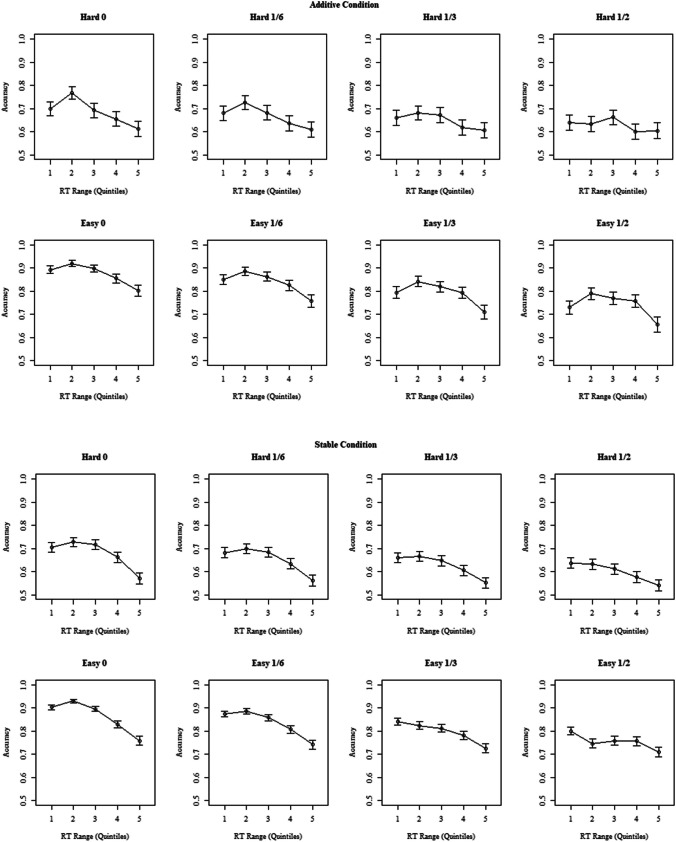
Conditional accuracy functions for Experiment 1. The figures show conditional-accuracy functions of accuracy as a function of response time (RT) for hard and easy trials in both the additive and the stable conditions. The first point on the x-axis represents the fastest responses (below the 20th percentile of RT), the second point the 20th to 40th percentile, etc. Error bars represent the standard error

Figure 4 shows a pattern consistent with the predictions of the doubt-scaling model: the overall level of the CAFs decreased as non-diagnostic information increased, and accuracy for slower bins increased relative to accuracy for faster bins, although the relative change was less marked for the hard additive condition.

## Discussion

Consistent with predictions based on the doubt-scaling model, the proportion of high-confidence responses – and participants’ ability to discriminate between correct and incorrect responses – decreased as non-diagnostic information increased. RT generally increased as non-diagnostic information increased, and CAFs showed that the accuracy of fast responses generally decreased relative to that of slow responses. The grid-type manipulation generally had little impact, with the exception of a larger difficulty effect on RT for stable than additive trials for the 1/6 proportion of non-diagnostic information, and a stronger effect of the proportion of non-diagnostic information on confidence in the stable condition than in the additive condition. The latter result appears to indicate an effect of the absolute amount of diagnostic information in addition to an effect of non-diagnostic information. Although the effect is only small (a reduction of 33% in the stable condition compared to a reduction of 28% in the additive condition), it suggests a slight deviation from the predictions of the doubt-scaling model. We note that this inconsistency was not evident in either accuracy or RT (aside from the three-way interaction with difficulty and non-diagnostic information). We observed response bias in the data, with participants demonstrating a bias towards orange at low levels of non-diagnostic information, switching to a bias towards blue at higher levels of non-diagnostic information. This may be due to the colours used in the dynamic grid, as there is a smaller difference between the RGB values of blue and white than between orange and white, making white more confusable with orange than blue.

## Experiment 2

Having participants consider confidence while making decisions slows the decision-making process (Baranski & Petrusic, 2001; Petrusic & Baranski, 2003). To test whether the observed effects of non-diagnostic information on accuracy and RT generalize, Experiment 2 removed the confidence ratings. Our hypotheses with respect to accuracy, RT, and their combination in CAFs remain the same.

### Method

#### Design

We used a 4 (proportion of non-diagnostic information: 0, 1/6, 1/3, ½) × 2 (difficulty: easy or hard) within-subjects design. The outcome variables were accuracy (*d*’) and RT (as indexed by CAFs and mean RT).

#### Participants

Twenty-one participants completed the experiment, with one being removed for truncated RTs. Renumeration and exclusion criteria were the same as Experiment 1.

#### Procedure

The procedure and materials were identical to those of Experiment 1, with three exceptions. First, there were only two response options (‘Orange’ and ‘Blue’). Second, as grid-type did not moderate the effects of non-diagnostic information on accuracy or RT, we used only the stable manipulation. Third, participants completed only two practice blocks before beginning the experimental trials (20 trials without the presence of non-diagnostic information, 40 trials with the non-diagnostic manipulation) as confidence ratings were no longer relevant.

#### Analysis methods

Data were analyzed using the same approach as Experiment 1.

## Results

### Discrimination

As expected, *d´* decreased significantly as non-diagnostic information increased, χ^2^(3) = 244, *p* < .001. Again, there was a main effect of difficulty, with participants showing better discrimination for easy than for hard trials, χ^2^(1) = 448, *p* < .001, and the effect of non-diagnostic information was weaker for the hard condition than for the easy condition, χ^2^(1) = 17, *p* < .001 (see Fig. 4, panel A). Figure 4 also shows that, in contrast to Experiment 1, discrimination was highest in the control (no non-diagnostic information) condition.

### Reaction times

**Fig. 5:**
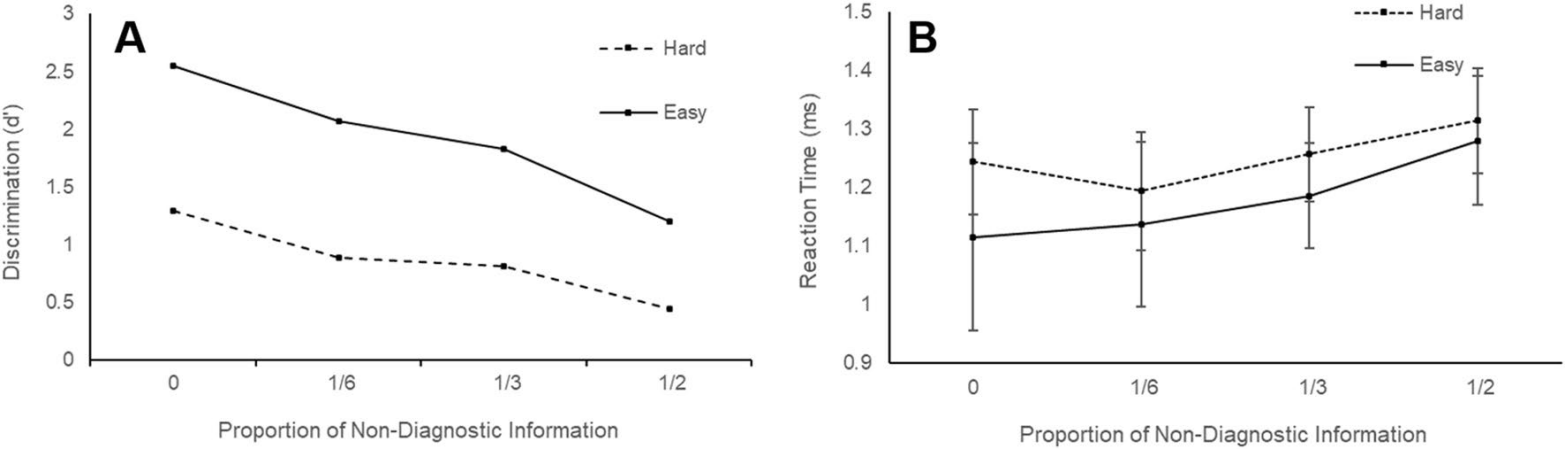
The effects of non-diagnostic information on accuracy (d*´*) and response time (RT) for Experiment 2. The figures demonstrate the relationship between non-diagnostic information and accuracy (as indexed by d´, panel **A**) and RT for all responses (panel **B**). Error bars represent the standard error

As per Experiment 1, a linear mixed-effects model on log RTs showed that RT generally increased as the proportion of non-diagnostic information increased, *F*(3,14347) = 90.5, *p* < .001. There was the expected overall slowing for difficult choices, *F*(1,14347) = 135.6, *p* < .001, which interacted with proportion of non-diagnostic information, *F*(3,14347) = 7.7, *p* < .001. The interaction was due to the effect of non-diagnostic information being weaker for the hard condition than for the easy condition, and the slowing in the control condition relative to the low non-diagnostic information condition seen in Experiment 1 was weakened (see Fig. 5, panel B).

### Conditional accuracy functions

**Fig. 6:**
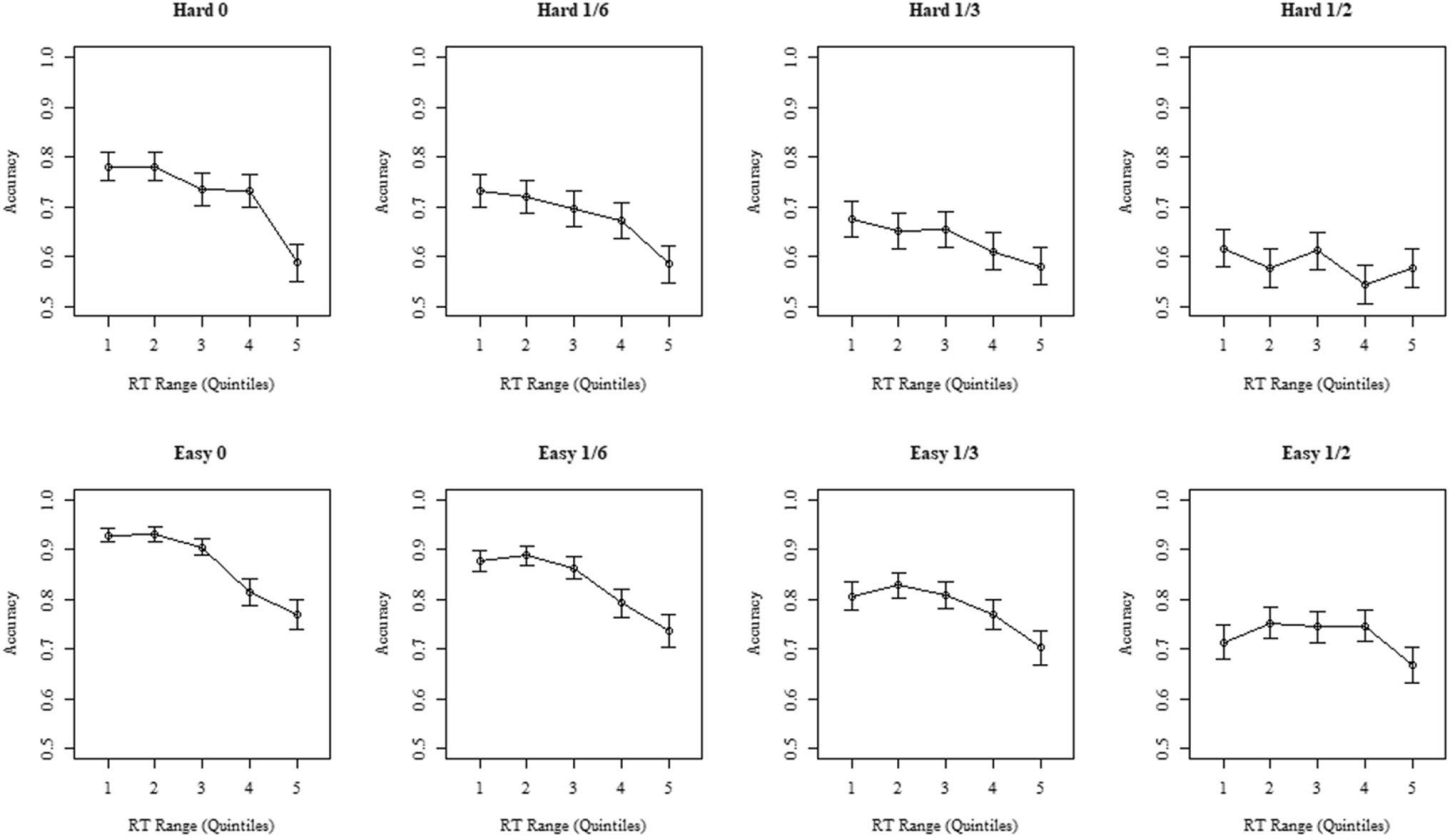
Conditional accuracy functions for Experiment 2. The figures show conditional accuracy functions as a function of RT for hard and easy trials in both the additive and the stable conditions. The first point on the x-axis represents the 20th percentile of RT, the second point the 20th to 40th percentile, etc. Error bars represent the standard error

Like Experiment 1, CAFs generally decreased when there was no non-diagnostic information and flattened as the amount of non-diagnostic information increases. Correspondingly, the interaction between RT range as factor and proportion of non-diagnostic information was significant, χ^2^(1) = 41.9, *p* < .001. In this case, the three-way interaction with difficulty was also significant, χ^2^(1) = 15.3, *p* = .004, reflecting stronger flattening in the easy condition (see Fig. 6).

## Discussion

The results of Experiment 2 support the generality of the predictions made by the doubt-scaling model with respect to accuracy and RT. First, discrimination decreased significantly as non-diagnostic information increased, while RT increased as non-diagnostic information increased. The effect on these measures was more monotonic than in Experiment 1, although the control condition was still slower than the low non-diagnostic condition for hard choices. Second, as non-diagnostic information increased, CAFs flattened, suggesting that participants’ errors sped up as the proportion of non-diagnostic information increased.

Again, we found significant interactions between difficulty and non-diagnostic information for effects on both accuracy and RT: the effect of the difficulty manipulation was less pronounced as the proportion of non-diagnostic information increased.

## General discussion

Baranski and Petrusic’s (1998) doubt-scaling model provides a framework for understanding the effect of non-diagnostic information (i.e., information that is not relevant to making a correct decision) on choice tasks. Although the model was proposed in the literature in 1998, it has not, to our knowledge, been directly tested in an experimental setting. Experiment 1 confirmed the model’s prediction that confidence decreases monotonically as non-diagnostic information increases, and that this holds for both harder and easier decisions. Both our experiments confirmed the prediction that accuracy decreases, and RT increases, as non-diagnostic information increased. However, results deviated from the prediction that the control condition should show the highest accuracy and lowest RT in Experiment 1 (where confidence judgements were required), and regarding RT for hard decisions in Experiment 2 (without confidence).

We also tested two more fine-grained predictions of the doubt-scaling model. First, it predicted that as non-diagnostic information increased so would the accuracy of slow responses relative to the accuracy of fast responses. In both experiments we confirmed this prediction, consistent with the idea that the presence of more non-diagnostic information causes faster and more frequent guessing responses. The second prediction concerns the effect of non-diagnostic information being determined by only the proportion rather than the absolute amounts of diagnostic and non-diagnostic information. This prediction held for accuracy and RT, but not for the confidence judgements in Experiment 1, where an increase in the proportion of non-diagnostic information had a stronger effect when it was accompanied by a decrease in the absolute amount of diagnostic information. Apart from these relatively minor deviations from predictions, our results provide clear support for the doubt-scaling model, at least in the context of the perceptual decisions studied in the present experiments. We now discuss the applied and theoretical implications of our results.

Although the present results are generally consistent with the predictions of Baranski and Petrusic’s (1998) doubt-scaling model, those predictions are not necessarily unique to that model. For example, the effect of non-diagnostic information can be framed as a manipulation of choice difficulty. Like typical difficulty manipulations, increasing non-diagnostic information reduces accuracy, increases RT, and reduces confidence. Choice difficulty is typically manipulated by reducing the difference between stimulus information favouring one choice versus stimulus information favouring the other choice. Hence, it is possible that non-diagnostic information affects decisions by reducing the subjective diagnosticity of information in the choice stimulus, even when, as our experiments demonstrate, objective diagnosticity is maintained in both a proportional and an absolute sense. One possibility is that non-diagnostic information acts as a source of noise, and so reduces the signal-to-noise ratio for a stimulus. A related possibility is that non-diagnostic information weakens a participant’s ability to selectively attend to diagnostic information, as in the account of Manley et al. (2019), again increasing the signal-to-noise ratio. Our experiments cannot rule out accounts like these where the effect of non-diagnostic information is on early processing stages rather than on a later response-selection stage as proposed by the doubt-scaling model.

However, such early-stage accounts would need to be elaborated to make testable in terms of specific predictions. Our finding that the CAF slope decreases may be particularly constraining such elaborations when an increase in non-diagnostic information is modeled by effects on the rate at which evidence about a choice is accumulated. For example, in the DDM a decrease in discriminability due to increase in rate variability leads to slower errors, which corresponds to CAF flattening (i.e., a greater proportion of errors are slow). In contrast, a decrease in discriminability due to an increase in the mean rate of evidence accumulation has less effect on error speed. However, evaluating these possibilities will likely require explicit model fitting. Another potential alternative explanation for the doubt-scaling account that was recently proposed by Hellmann et al. (2023) combines early and late approaches. Their model accumulates evidence about stimulus discriminability that is combined with evidence accumulated about a choice to determine confidence. This model could potentially be applied to explain the results of our first experiment. Although we saw effects of non-diagnostic information in the absence of confidence judgements in our second experiment, it is possible that their model could be extended to account for this case as well. We did find some deviations from the predictions of the doubt-scaling model that suggest some avenues for further theoretical investigation. First, the effect of the absolute amount of diagnostic information is consistent with the small but reliable effects of absolute stimulus magnitude found in paradigms ranging from simple perceptual choices (Teodorescu et al., 2016) to value judgements (Miletić et al., 2021). Modern descendants of Audley’s (1960) runs model, such as Usher & McClelland (2001) Leaky Competitive Accumulator model and van Ravenzwaaij et al.’s (2020) Advantage Linear Ballistic Accumulator model, produce small magnitude effects as the relative amount of information for each choice increases. In future work, it would be interesting to apply these models to manipulations of non-diagnostic information.

A second potential theoretical extension could be considered with respect to the doubt-scaling model’s guessing mechanism. Hawkins and Heathcote (2021) used a guessing process to provide a broad and integrative account of the effect of the passage of time on decisions. Like the doubt-scaling model, their Timed Racing Diffusion Model (TRDM) produces guesses when a third accumulator beats both accumulators that accrue diagnostic information about a binary choice. In their case, the guessing accumulator is driven by a constant input, and so provides a measure of the passage of time. It would be interesting to investigate whether the rate of this accumulator is modulated by the presence of non-diagnostic information, further broadening the explanatory reach of the TRDM.

In a more applied vein, our work also raises questions regarding the mechanisms that lead to higher accuracy rates for recognition tasks when lineup members are presented simultaneously versus sequentially. It has been suggested that simultaneous presentation allows participants to discount features that are shared by the lineup members and hence non-diagnostic (Wixted & Mickes, 2014; Wixted et al., 2018). Although discounting of common features seems likely to occur at least to some degree with faces and other complex visual stimuli (see also Heathcote et al, 2009; Tulving, 1981), our findings suggest that the presence of non-diagnostic information could have a residual effect in terms of decreased confidence and accuracy. Hence, further work examining the role of non-diagnostic information on decision making using a complex recognition task (compared with a basic perceptual task) is needed to clarify the generalizability of our results. Another question raised by this study pertains to whether we would expect the same pattern of results if the participants believed the non-diagnostic information to be diagnostic to the decision. While we cannot answer this question with our existing experimental paradigm, it highlights an avenue for further research.

In conclusion, given the fact that non-diagnostic information has the potential to be present in a variety of important applied decision-making scenarios (e.g., identifying a weapon within a crowded piece of luggage, or recognizing a tumour in a medical x-ray), a greater focus on its effects seems warranted. The current research provides some initial insight into the effects of non-diagnostic information that may be utilized in applied scenarios to better understand and evaluate decision making. In future work it would be useful to extend our investigation to the sorts of complex perceptual decisions, and recognition memory decisions, relevant to these applied contexts, as well as by developing and quantitatively evaluating detailed models.

## Data Availability

Raw data and analyses codes for both Experiment 1 and Experiment 2 are available via the Open Science Framework at https://osf.io/exqba/ Contact the corresponding author for a copy of the experimental code.
